# Dr. Friese, on Vaccination

**Published:** 1807-01

**Authors:** John Ring

**Affiliations:** New Street, Hanover Square


### *To the Editors of the Medical and Physical Journal.*

GENTLEMEN,

FROM my last communication it appears, that the slothful Spaniard has carried the blessings of vaccination round the globe, before Great Britain, where the discovery was made, has determined whether it is worth while to adopt the practice at home, except in her Army and Navy. It is true, John Bull has made an acknowledgment to the author of the discovery, and is doubtful whether he has done him justice; but in other respects, he has slumbered over the subject seven years; and is only now beginning to inquire seriously, whether it is worthy of being considered as a national concern.

His sentiments concerning this practice are rather singular. Laws may be enacted to check the ravages of the plague; but no laws must be enacted to check the ravages of the small-pox. It still remains to be seen, whether, in this instance alone, popular clamour is to prevail over reason; whether a salutary measure may be promoted by the authority of the state; whether the most destructive pestilence may be propagated with impunity, and whether the only liberty which must not be abridged, for the sake of the community at large, is the liberty of doing mischief.

It is evident from a great variety of documents, which I have already published in this and other channels, that Great Britain is almost the only nation in Europe, in which vaccination is not promoted by the direct interference and influence of government. In addition to other evidence of this kind, I shall here transcribe the substance of a third communication from Dr. Friese, Director General of Vaccination in Silesia, dated Aug. 14, 1806.

He states, that although several impediments had occurred to arrest the progress of vaccination in that department, such as the war raging in Germany, with all the evils naturally attendant on that scourge of mankind, yet the number of individuals secured from the small-pox by this prophylactic, in the course of 1805, amounted to 17316; which is 7003 less than in the former year. The small-pox broke out early in the spring, and prevailed more or less during the whole year; but those who had been vaccinated escaped its infection; affording sufficient proofs, to convince the most incredulous of the efficacy of the the practice. Some blunders had been committed there, as well as in other parts of the world; but Dr. Friese flattered himself they would not so far bring vaccination into discredit, as to prevent the number of inoculations in the present year from exceeding any of the past.

In spite of the calamities with which that country has been afflicted, there are several districts of Silesia, where, in consequence of the indefatigable endeavours of medical practitioners, vaccination has surmounted every obstacle; and manifested its happy effects in a most surprising manner. This was particularly evinced in the district of Olaw; where Dr. Wenzke had taken such measures for exterminating the small-pox, that almost all the children who had not undergone this disease were vaccinated. The consequence was, that although the small-pox was three times introduced by travellers, yet it was never able to gain ground; and only two children, who were born subsequently to the general vaccination, caught the disorder.

To prevent similar inconveniences for the future, and, if possible, to carry the practice of vaccination, in Silesia, far beyond its former extent, Dr. Friese was charged by the medical department, to propose to Government a plan for prosecuting this important object on a large scale. Government was graciously pleased to comply with his proposal; and the following regulations have been published by the Royal Chamber at Breslaw.

1. Every counsellor of a district shall assemble the inhabitants, and the magistrates, every quarter; in order to explain to them the great advantages of vaccination.

2. The more effectually to promote this laudable design, a popular publication, gratuitously distributed by Government, shall be read every quarter, in the presence of the inhabitants who are assembled, by the clerk or schoolmaster of the village.

3. If the small-pox breaks out in any village, the counsellor of the district shall instantly repair thither, attended by the physician and surgeon of the district; and insist on all children, who are susceptible of that disorder, being instantly presented for vaccination.

4. Should any parents be so obstinate as to refuse to adopt this practice, and any one of their children be, in consequence, infected with the small-pox, the counsellor of the district shall forthwith pursue proper measures, to prevent the disorder from spreading, by cutting off all communication between the infected family and the neighbourhood.

5. Should

5. Should the small-pox extend through a whole village, the aforesaid measures shall also be extended; and be executed exactly in the same manner, as in those places where the murrain breaks out among cattle; and be continued till it appears, by a testimonial of the attending physician, that all danger is over.

6. With regard to the burial of those who die of the small-pox, the restrictions which were published in a circular order, in the year 1788, shall be enforced with all possible rigour.

7. The same measures shall be pursued by magistrates, in towns and boroughs.

8. No child shall be admitted to any school, orphan-house, hospital, or any other public institution whatsoever, unless it be satisfactorily proved, that he has already undergone, or his parents or tutors consent that he shall undergo, vaccination.

9. Clergymen of all persuasions, shall avail themselves of every opportunity of stating to their parishioners, the benefits which may be derived from a general introduction of the new practice; and endeavour, by all the means in their power, to eradicate the prejudices of the people.

In addition to this plan, and in order to excite the greater emulation, Government has granted the same premiums as in the year 1804, to several clergymen, physicians, and surgeons, who have distinguished themselves in this line.—The Royal Vaccine Institution established at Berlin, has continued its labours with uniform success.

Dr. Friese next makes the following remarks on our late proceedings in this country. "I see, by the Medical and Physical Journal, that the contest concerning the value and efficacy of vaccination, is still carried on in England with the utmost vigour. I have attentively perused all the publications of Messrs. Goldson, Moseley, Squirrel, Birch, Rogers, Rowley, and several other adversaries of the Jennerian discovery; but I am dissatisfied with all their ill-founded arguments and facts.

"I am concerned to see, how far a character may be degraded by partiality, jealousy, and self-interest, as in the instance of Dr. Rowley; whose virulence far exceeds that of his prototypes in France and Germany; and even that of his insolent fraternity in England. In a letter to my friend Dr. De Carro, I once nicknamed Dr. Ehrmann of Frankfort, *the Antivaccinarian Marat*; but now I clearly perceive that the president of the London Antivaccinarian Society has a more just claim to the title.

"For

" For my own part, I assure you upon my honour, that I have never met with a single case of either of the four newly-discovered cow-pock maladies, the frightful names of which adorn the title page of the Doctor's incomparable work. Time and experience will detect these impositions.

" Permit me to advert to another question relative to vaccination. I am entirely of your opinion when you admit, that cases of small-pox after cow-pox may sometimes, though but rarely occur; yet I do not see how the antivaccinists can hence draw any argument against the practice; since it is a well-known fact, that there are also some instances attested by the best authorities, in which the small-pox has occurred a second time, in those who had long before had the disorder by inoculation, or in the natural way.

" There is no rule without an exception; and if such exceptions seem to be more frequent than before, it is because the antivaccinists now declare every kind of eruption which appears on children who have been vaccinated to be the small-pox, in order to accomplish their knavish design. I have often met with instances, in which it was extremely difficult for the most able practitioner to distinguish the chicken-pox from the small-pox; and I was a witness to one case, in which a very respectable physician of this city, inoculated three children of a merchant still living at Berlin, with varicellous instead of variolous matter. They were all pronounced to be secure; but the next year they all had a heavy crop of the small-pox, and with difficulty survived.

" I am anxious to see your refutation of Moseley; of which I know the existence only by the Medical and Physical Journal. I hope, with Aculeus, in his Letters to Dr. Rowley, " a time will come, when the firm supporters of the cause will look back with heartfelt satisfaction on their past labours; and receive the reward of public confidence, and public gratitude, which they so richly merit, for their disinterested zeal and benevolent perseverance; while the insects of opposition shall be forgotten, or only be remembered on account of the trouble which they have occasioned."

The next article which I shall bring forward, is one, which will shew, in a striking point of view, the different estimation in which the Jennerian discovery is held in one of the most valuable parts of the British dominions. It is an extract of a letter, dated April 25, 1806, which I some time time ago received from Mr. Weston, of St. Ann's Bay, Jamaica; whose former correspondence I have already published in this Journal.

"I have withheld my acknowledgments for your goodness, in regularly supplying me with vaccine matter, with the vain hope, that with my thanks, I should be enabled to communicate the important information, of vaccination being universally established in this island. But, alas! a combination of scepticism and indolence have prevailed so far, as to render it only a *partial* good.

"I have repeatedly succeeded with matter obtained from you, and from the continent of America; and, were I capable of rousing my brethren to a just sense of the value of vaccination, we should have it in a state of activity. Though I have succeeded in introducing it into Kingston, and the neighbouring parishes; yet, for want of zeal, its continuance there has been very transient.

"Within my circle I have thrice introduced it; and each time been obliged to desist for want of subjects. On the whole I have vaccinated about nine hundred persons; fifty of whom I have submitted to the test of variolous contagion, both by puncture and inhalation; but without producing any but a slight local effect.

"A few months ago, a servant of mine, whom I supposed to have had the small-pox, was seized with that disorder, in the confluent way, so that the surface of his body was one entire mass of matter. While he was in this state, I exposed three children of my own family, whom I had vaccinated with virus obtained from you, in the early part of 1803, to the infection in various ways; but the blessed prophylactic had rendered them invulnerable to the fatal scourge.

"I smile when I read, that the pompous Dr. Moseley has inoculated thousands of people in the West Indies with the small-pox without losing a patient. Dr. Moseley practised at Kingston, in this Island; and it is a well-known fact, that his practice was extremely limited, and that he was much more devoted to music than to medicine."

Dr. Moseley observed, that he spoke subject to the correction of his contemporaries. I have on a former occasion corrected the mistake which he committed, when he asserted that he had never lost a patient by inoculation; and Mr. Weston here corrects another. Many people wonder how Doctor Moseley, the Physician of Chelsea Hospital, came to dabble with inoculation; but their wonder will cease cease when they are informed, that when he was in the West Indies, he moved in the humble sphere of an apothecary.

Mr. Weston concludes as follows. "I have resided in this country almost twenty years, and have, in the course of that time, inoculated about three thousand people; but so far from being as lucky as Dr. Moseley, I have lost, on an average, one in a hundred and fifty; and have no reason to think that I was more unsuccessful than my neighbours.

"To you, whose indefatigable labours, in the cause of suffering humanity, do you so much honour, I am indebted for the means, which have enabled me to rescue a few of my fellow creatures from the impending evil of the small-pox. Fervently wishing, that you may proceed in your benevolent career, unchecked by ignorance or self-interest, until your humane endeavours are crowned with success, I remain, dear Sir, very respectfully,

"Your obliged and obedient servant,

"Wm. Weston."

To this account of the state of vaccination in the West Indies, I beg leave to subjoin an extract of a letter from Dr. Spalding, of Portsmouth in New England, to Doctor Jenner, dated May 27, 1805.—"Be pleased to accept my *Portsmouth Bills of Mortality for* 1803 *and* 1804.—The blessings of the cow-pock spread far and wide in this country; and, since the introduction of the practice, the small-pox is unknown among us. We have now no unbelievers; all are satisfied."

This general conviction of the efficacy of vaccination, which Dr. Spalding represents as prevailing in his neighbourhood, must, in a great measure, be ascribed to his zeal; which is such as might well be expected from a pupil of Waterhouse.—Having had the pleasure of being acquainted with him while in England, I am happy in reflecting, that the expectations which I then formed of him have not been disappointed.

The last evidence which I shall here adduce, to shew the present state of vaccination, is that from France and Italy. The latest reports from both these kingdoms, abundantly prove, that our enemies know how to appreciate the blessings of that divine art, much better than the people of this country, with all their boasted philosophy and good sense. This information therefore, I trust, will act as a seasonable reproof; and a powerful incentive to fresh exertions.

In

In France, four hundred thousand persons have been vaccinated within the last year; and the following substance of a letter from Doctor Sacco, of Milan, Director General of Vaccination in the Kingdom of Italy, to Dr. Odier of Geneva, dated Milan, Jan. 23, 1806, and published in the Bibliotheque Britannique, bears ample and honourable testimony, that Italy vies with France in her endeavours to exterminate the most dreadful scourge of the human race.

Dr. Sacco having been sent for, in September, 1805, to Florence, where the small-pox was epidemic, in order to vaccinate a child of M. Tassoni, Charge d'Affaires of His Imperial Majesty at the Court of Hetruria, vaccinated a considerable number of persons, in the presence of the principal physicians and surgeons of that city; and afterwards put to the test of variolous inoculation, not only those whom he had recently vaccinated, but also those who had been vaccinated two, three, and four years before by the medical practitioners of that city.

All these experiments were crowned with complete success. The Queen of Hetruria, to whom Dr. Sacco had the honour of being introduced, presented him, on this occasion, with a very beautiful gold medal; on one side of which are the figures of Her Majesty and the young King; and on the other, the following inscription, Maria Louisa, Queen Regent, to Dr. Sacco.

From Florence he went to Parma, where he remained only four days; nevertheless, being seconded by the zeal and influence of M. Moreau St. Mery, he vaccinated four hundred persons. On his return to Milan, *from which place the small-pox had been banished for the space of three years, by means of vaccination*, he found that it had been introduced there once more, by a person arrived there from Florence. It quickly began to spread in the neighbourhood; *but as there is a law ordaining, that information should be given of any persons who are attacked with the disorder*, only seven had it in the city. *All the rest, amounting to twenty-six, were carried to a lazaretto; and kept in a state of seclusion.*

In the mean time *a general inoculation of the cow-pock was commenced, both in town and country.* The number of persons vaccinated on this occasion, *amounted to nearly* 5000; *and was daily increasing, at the rate of from* 130 *to* 160 *a day. By these means the small-pox was again speedily exterminated.* Fas est et ab hoste doceri.

I am, &c.

JOHN RING.

*New Street, Hanover Square.*

